# Structural insights into substrate and inhibitor binding sites in human indoleamine 2,3-dioxygenase 1

**DOI:** 10.1038/s41467-017-01725-8

**Published:** 2017-11-22

**Authors:** Ariel Lewis-Ballester, Khoa N. Pham, Dipanwita Batabyal, Shay Karkashon, Jeffrey B. Bonanno, Thomas L. Poulos, Syun-Ru Yeh

**Affiliations:** 1 Department of Physiology and Biophysics, Albert Einstein College of Medicine, Bronx, NY 10461 USA; 2 Department of Molecular Biology and Biochemistry, University of California, Irvine, CA 92697 USA; 3 Department of Pharmaceutical Sciences, University of California, Irvine, CA 92697 USA; 4 Department of Chemistry, University of California, Irvine, CA 92697 USA; 5 Department of Biochemistry, Albert Einstein College of Medicine, Bronx, NY 10461 USA

## Abstract

Human indoleamine 2,3-dioxygenase 1 (hIDO1) is an attractive cancer immunotherapeutic target owing to its role in promoting tumoral immune escape. However, drug development has been hindered by limited structural information. Here, we report the crystal structures of hIDO1 in complex with its substrate, Trp, an inhibitor, epacadostat, and/or an effector, indole ethanol (IDE). The data reveal structural features of the active site (Sa) critical for substrate activation; in addition, they disclose a new inhibitor-binding mode and a distinct small molecule binding site (Si). Structure-guided mutation of a critical residue, F270, to glycine perturbs the Si site, allowing structural determination of an inhibitory complex, where both the Sa and Si sites are occupied by Trp. The Si site offers a novel target site for allosteric inhibitors and a molecular explanation for the previously baffling substrate-inhibition behavior of the enzyme. Taken together, the data open exciting new avenues for structure-based drug design.

## Abstract (web summary)

Human indoleamine 2,3-dioxygenase 1 (hIDO1) is an immunotherapeutic target for cancer therapy. Here, the authors present the substrate-, inhibitor- and effector-bound hIDO1 crystal structures, which give insights into the mechanism and reveal a second small molecule binding site, which is of interest for drug design.

## Introduction

hIDO1 is a heme-containing enzyme that catalyzes the first and rate-limiting step of the kynurenine pathway—the dioxygenation of Trp to N-formyl kynurenine (NFK)^1–3^. It negatively regulates cellular immune response via depleting Trp and promoting the accumulation of kynurenine pathway metabolites^4–7^. Increased expression of hIDO1 is associated with poor prognosis and shortened survival of cancer patients^8,9^. An analog of hIDO1, human tryptophan dioxygenase (hTDO)^10^, and a second isoform of hIDO1, named hIDO2^11,12^, have also recently been identified as immunomodulatory proteins with potential relevance to cancer. Together, they represent a new attractive class of immunotherapeutic targets. Intense efforts have been devoted to develop inhibitors against the three proteins^13^. So far, all the reported hIDO1 inhibitors target the active site, Sa, which is spacious and flexible; in addition, most of the inhibitors are prone to heme iron coordination. Together, they pose serious limitations in computer-aided drug design^13^. It is hence important to have a comprehensive understanding of the structural properties of hIDO1. The structures of hTDO have been solved in both substrate and product-bound states^14^, while those of hIDO1 are limited to substrate-free forms^15–18^. Here we report the structures of the wild-type hIDO1 in complex with Trp, an inhibitor (epacadostat), and/or an effector (IDE), as well as comparable structures of an active site mutant, F270G. The data shed new light into substrate–protein and inhibitor–protein interactions in the distal heme pocket; in addition, they unveil a small molecule binding site in the proximal heme pocket. The implication of the data on the dioxygenase and substrate-inhibition mechanisms of hIDO1, as well as structure-based design of hIDO1-selective inhibitors, will be discussed.

## Results

### Binding mode of Trp

**Fig. 1:**
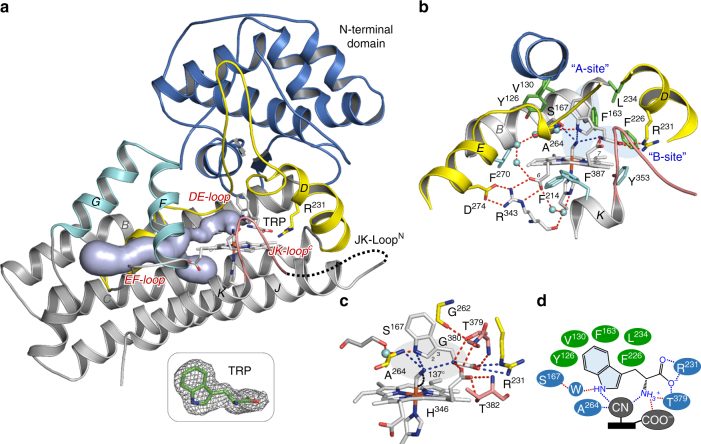
Binding mode of Trp. **a** Crystal structure of the hIDO1-CN-Trp complex. The nomenclature of the α-helices is based on sequence alignment shown in Supplementary Fig. 1. The black dotted line indicates the disordered JK-Loop^N^. The tint lightblue surface illustrates an active site access tunnel that penetrates through the EF-Loop, along one side of the E-Helix, towards F270 in the E-Helix (not shown), where it bifurcates into two branches reaching out to the distal and proximal heme pockets. The tunnel is contoured with the Caver 3.02 plugin in PyMol (http://caver.cz/). The 2Fo-Fc map of the bound Trp shown in the bottom inset is contoured at 1.0 σ. **b**,** c** Blow-up views of the active site, Sa. The Trp binds in the distal heme pocket, with the indole ring occupying the A site and the carboxylate/ammonium groups occupying the B site, as highlighted in lightblue background in **b**. The Trp interacts with the protein matrix, as well as the heme, via various hydrophobic and polar interactions as summarized in **d** (see details in the main text). Together, they position the terminal atom of the diatomic ligand next to the C_2_ of the Trp in an oxyanion hole, highlighted in grey background in **c**; in addition, they distort the porphyrin macrocycle out of the typical planar conformation and produce an imidazolate character on H346, both of which are critical for the dioxygenase activity of the enzyme^3^. It is noted that T379 and G380 are part of the “GTGG” motif in the JK-Loop^C^, which is fully conserved in IDO1 and TDO families of enzymes (Supplementary Fig. 1), while G262 and A264 are part of the DE-Loop

As shown in Fig. 1a, hIDO1 is an α-helical protein, made up by a large C-terminal domain containing the Sa site and a small N-terminal domain sitting on top of it. Trp binding in the Sa site induces the organization of the C-terminal fragment of the highly disordered JK-Loop (referred to as the JK-Loop^C^ hereafter) into a β-hairpin structure, while the N-terminal fragment of the loop (referred to as the JK-Loop^N^ hereafter) unexpectedly remains disordered. The JK-Loop^N^ is only present in hIDO1, not in hTDO, although the two dioxygenases share high structure-based sequence homology, in particular in the active site (Supplementary Fig. 1). The JK-Loop^N^ contains two phosphorylation sites^19^; in addition, the truncation of the loop fragment by up to 14 residues only slightly reduces the enzyme efficiency by approximately fourfold, suggesting that the biological function of the highly flexible JK-Loop^N^ is to control signal transduction, rather than regulating enzyme activity. Other than the JK-Loop^N^, several unique structural features of hIDO1 distinguishing it from hTDO are identified as discussed in the Supplementary Fig. 2.

The substrate Trp binds in the distal heme pocket, which is enclosed by the JK-Loop^C^ and the DE-Loop, and is capped by a small helix from the N-terminal domain. The binding site is lined with a group of hydrophobic residues, including F226, L234, F163, V130, and Y126 (Fig. 1b). The indole group of the Trp, lying perpendicular to the heme, occupies the so-called “A site”^13^. Its indoleamine group H-bonds with S167 via an intervening water molecule. The ammonium and carboxylate groups extend into the so-called “B site”^13^, forming an interlaced H-bonding network with the conserved “GTGG” motif in the JK-Loop^C^ (Supplementary Fig. 1) and the G262 in the DE-Loop, as well as the 7-propionate group of the heme (Fig. 1c). The carboxylate group is further secured by ion-pairing with R231, which plays a critical role in substrate binding and recognition. Together, these interactions force the iron-bound CN to adopt an unusual bent conformation (with the Fe–C–N angle of ~137°) and position its nitrogen atom next to the C_2_ atom of the Trp in an oxyanion hole, made up by the indoleamine and ammonium groups of the Trp and the peptide amide group of A264. The active site structure is distinct from that of hTDO (Supplementary Fig. 3), but it resembles that of the hIDO1-O_2_-Trp complex predicted by QM/MM simulations^3,20–22^, offering a direct evidence supporting the hypothesized radical addition mechanism (Supplementary Fig. 4). The Trp-binding site is completely shielded from the bulk solvent, except that a tunnel penetrating from bulk solvent into the distal and proximal heme pockets is evident (displayed as the tint lightblue surface in Fig. 1a). Although the tunnel is relatively narrow, dynamic motion of the residues lining the tunnel may allow it to function as a conduit for delivering small molecules into or out of the Sa site and the Si site (which will be defined later).

### Binding mode of epacadostat

**Fig. 2:**
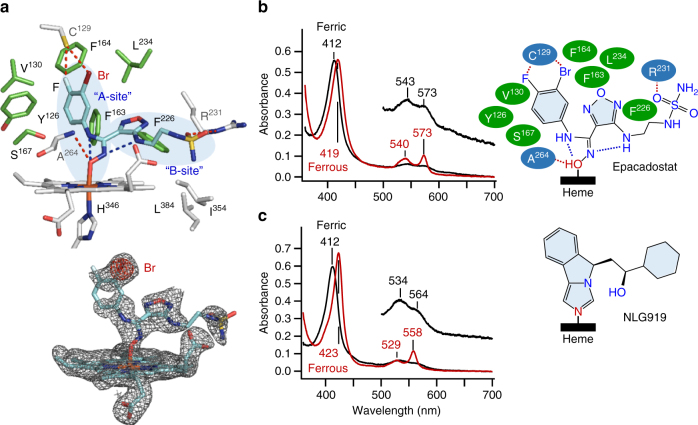
Binding mode of epacadostat and spectral markers for O and N-based inhibitors. **a** Crystal structure of the hIDO1-epacadostat complex. The interactions between epacadostat and the protein matrix are indicated by the red dotted lines, while the intramolecular H-bonds within epacadostat are indicated by the blue dotted lines. The bottom inset shows the 2Fo-Fc map of the epacadostat and the heme, contoured at 1.0 σ (colored in gray). It is overlaid with the anomalous difference Fourier map contoured at 9.0 σ (shown in red), confirming the position of the Br atom. **b**,** c** Optical absorption spectra of hIDO1 in complex with epacadostat or NLG919, which coordinates to the heme iron via its oxygen or nitrogen atom, respectively, in the ferric state (black trace) and ferrous state (red trace). The right inset in **b** shows a schematic illustration of the epacadostat–protein interactions. It should be noted that the bound epacadostat is displayed as a protonated form, but its true protonation state requires additional studies

We next crystalized hIDO1 in complex with epacadostat, the most advanced hIDO1 inhibitor in clinical trials^23^, and determined its structure at a resolution of 2.50 Å. Modeling of the inhibitor in the Sa site was assisted by identification of a peak consistent with Br in the anomalous difference Fourier electron density map (see Methods section). The data reveal that epacadostat coordinates to the heme iron via the oxygen atom of the hydroxyamidine group (Fig. 2a). The unusual O-based coordination bond has not been experimentally identified in any known hIDO1-inhibitor complex structures, although it has been suspected based on molecular docking simulations^23^. The coordination bond is stabilized by a H-bond with A264 in the DE-Loop, as well as two intramolecular H-bonds within the inhibitor, as summarized in the inset in Fig. 2b. The benzene group occupies the “A site”, and is surrounded by a group of hydrophobic residues, including F163, L234, F164, V130, Y126, and S167. The Br and F atoms sit next to C129 in the N-terminal domain, offering a favorable fluorine–sulfur contact that is known to stabilize protein–inhibitor interactions^24^. The side arm of epacadostat extends out into the “B site”, with the furazan group stabilized by F163, L234, and F226, and the sulfamide group stabilized by R231. To determine the electronic properties of the hIDO1-epacadostat complex, we carried out optical absorption spectroscopic studies. The spectra of the ferric and ferrous complexes show peak maxima at 412/543/573 nm and 419/540/573 nm, respectively (Fig. 2b). They are similar to those of the complexes with INCB149439, an analog of epacadostat (Supplementary Fig. 5D), but distinct from those of the complexes with N-based inhibitors that coordinate to the heme iron via the nitrogen atom of their imidazole groups, such as NLG919 (Fig. 2c and Supplementary Fig. 5B) or 4-phenylimidazole (Supplementary Fig. 5C), which exhibit characteristic peak maxima at 412/534/564 nm and 423/529/558 nm, respectively. The unique spectral markers of the O-based and N-based inhibitors provide useful guidelines for future evaluation of new hIDO1 inhibitors.

### A second small molecule binding site in hIDO1

**Fig. 3:**
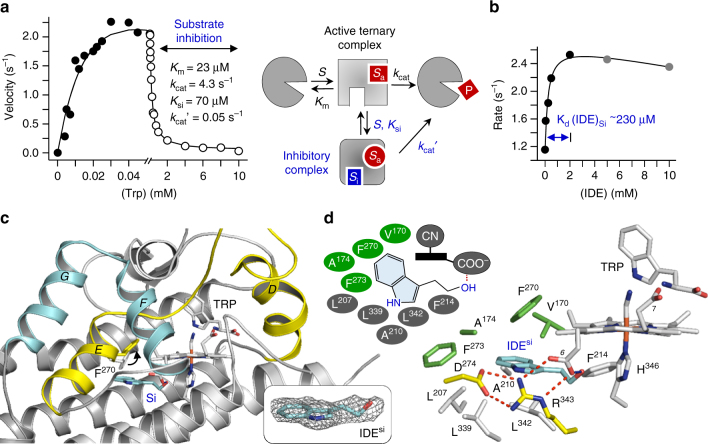
Identification of a second small molecule binding site (Si) in hIDO1. **a** Steady-state activity of hIDO1, showing the substrate-inhibition behavior at [Trp] >40 μM. The right panel shows a previously proposed two Trp-binding sites model^8^ suggesting that, at low [Trp], the substrate binds to the active site (Sa) to generate the active ternary complex, which can turn over to produce N-formyl kynurenine (NFK); while at high [Trp], a second Trp binds to an inhibitory site (Si) to generate the inhibitory complex, which exhibits impeded activity due to an allosteric structural transition to the Sa site. The kinetic parameters derived from the best fit of the data with the model are indicated in the plot. **b** Activity of hIDO1 as a function of [IDE], showing the function of IDE as an effector by binding to the Si site. It is notable that, at high concentrations (>2 mM), IDE can compete with Trp for the Sa site, leading to inhibited activities (see the data points labeled in gray). **c** Crystal structure of the hIDO1-CN-Trp complex in a mixed ligand state, where the Sa site and the Si site are occupied by Trp and IDE, respectively. The F270 side chain colored in gray, taken from the IDE-free structure (Fig. 1), is shown as a reference. The upward movement of the F270 side chain induced by IDE binding to the Si site is indicated by the black arrow. The bottom right inset shows the 2Fo-Fc map of IDE contoured at 1.0 σ. **d** Blow-up view of the IDE-binding site. The hydrophobic residues forming the base of the binding pocket are shown as gray sticks, while those lining the top of the pocket are shown as green sticks. The upper left inset shows a schematic illustration of the IDE-protein interactions

To explore additional inhibition site(s), we used IDE (3-indole ethanol) as a structural probe. IDE is an effector of hIDO1^25^. Earlier studies suggest that IDE promotes enzyme activity by occupying a hypothesized second Trp-binding site, Si, which is responsible for substrate inhibition behavior of the enzyme (Fig. 3a)^25^. Activity studies as a function of [IDE] show that the *K*_d_ (IDE)_si_ is ~230 μM (Fig. 3b). We crystallized the hIDO1-CN-Trp complex in the presence of excess IDE, and determined its structure at a resolution of 2.60 Å (Fig. 3c). Electron density associated with IDE is surprisingly evident on the proximal side of the heme, offering the first glimpse of the long-suspected Si site. The IDE sits on top of a hydrophobic base constituted by L207, L339, A210, L342, and F214 (Fig. 3d). Its indole ring forms an offset *π*-stack with the heme, while its OH group establishes a H-bond with the 6-propionate group. The overall structure of the enzyme is mostly unaffected by IDE binding, except that the EF-Loop becomes slightly more rigid, while the JK-Loop^C^ is more flexible (Supplementary Fig. 6A, B). More importantly, significant conformational changes are evident in the E-Helix. In particular, the side chain of F270 is pushed up due to a steric clash with the indole ring of IDE. This finding prompted us to construct and study the F270G mutant, in which the bulky side chain is eliminated.

### Si site as an inhibitor-binding site

**Fig. 4:**
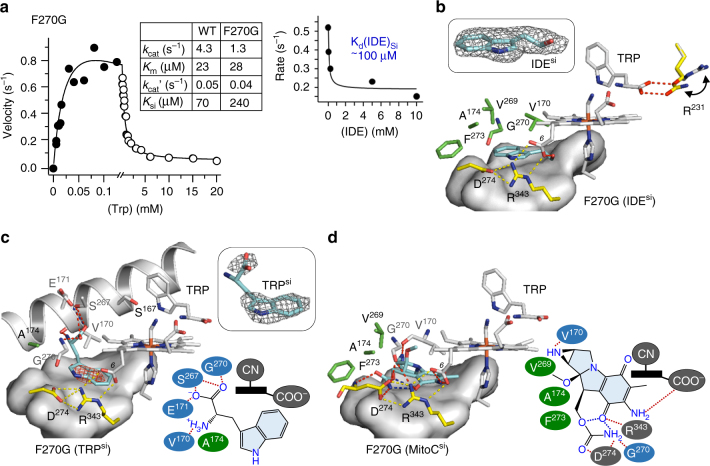
Evidence supporting the Si site as an inhibitor-binding site. **a** Steady-state activity of the F270G mutant of hIDO1. The kinetic constants obtained by fitting the data with the two-Trp-binding sites model are summarized in the table in the inset, where the wild-type enzyme data are listed as a reference. The right inset displays the activity of F270G as a function of [IDE]. In contrast to that observed in the wild-type enzyme, IDE binding inhibits the activity; in addition, only one IDE-binding event, with a *K*_d_ (IDE) of ~100 μM, was observed. The inhibition effect is attributed to IDE binding to the Si site, not the Sa site, based on two observations: (i) the *K*_d_ is similar to the *K*_d_ (IDE)_si_ of the wild-type enzyme (~200 uM), and (ii) the crystal structure shown in **b** demonstrates that, when IDE and Trp coexist, IDE preferentially occupies the Si site. **b** Crystal structure of the F270G-CN-Trp complex in a mixed ligand state, where the Sa and Si sites are occupied by Trp and IDE, respectively. The 2Fo-Fc map of the Si site IDE in the inset is contoured at 1.0 σ. The surface representation shows the hydrophobic base of the Si site built by L207, L339, L342, A210, and F214. **c** Crystal structure of the F270G-CN-Trp complex in a two Trp-bound state, where both the Sa and Si sites are occupied by Trp. The red mesh represents the simulated annealing omit map of the Si site Trp contoured at 1.0 σ. The top right inset shows the 2Fo-Fc map of the Si site Trp contoured at 1.0 σ. The bottom right inset shows a schematic illustration of the Trp-protein interactions in the Si site. **d** Structure of the F270G mutant in complex with mitomycin C (MitoC) obtained by molecular docking studies. The right inset illustrates the predicted MitoC-protein interactions

The F270G mutation leads to approximately fourfold reduction in *k*_cat_, while the *k*_cat_′ remains almost the same; in contrast, *K*_m_ is unaffected, while *K*_si_ is raised by approximately threefold (Fig. 4a). Titration studies surprisingly reveal that IDE functions as an inhibitor, instead of an effector (see the right inset). In addition, the apparent *K*_d_ (IDE) is reduced from 230 to 100 μM. We next crystallized the F270G-CN-Trp complex in the presence and absence of IDE, and solved their structures at a resolution of 2.69 and 3.03 Å, respectively. In the IDE complex (Fig. 4b), IDE binds to the Si site in a manner similar to that observed in the wild-type complex. Remarkably, in the absence of IDE, electron density associated with Trp is evident in the Si site (Fig. 4c). It demonstrates that the Si site can indeed accommodate a second Trp, offering molecular explanation for the substrate inhibition behavior of the enzyme. Why is Trp not observed in the Si site of the wild-type enzyme, in particular considering the fact that its *K*_si_ is approximately threefold lower than that of the mutant? It is important to note that the affinity of Trp towards the Si site is sensitive to the oxidation and coordination states of the heme iron^26,27^. The *K*_si_ values determined by the activity measurements plausibly reflects the Trp affinity towards the oxy complexes (which are preferentially populated during active enzyme turnover), not the ferric hIDO1-CN-Trp complex. The *K*_d_ (Trp) of the Si site in the ferric hIDO1-CN-Trp complex is too high (~26 mM)^27^ to allow the trapping of the second Trp in the wild-type crystals.

The F270G data show that the Trp bound in the Si site adopts a unique conformation with respect to IDE. Specifically, its indole group flips 180°, such that the ammonium and carboxylate groups point away from the proprionate-6 group of the heme. The carboxylate and ammonium groups are in position to H-bond with E171/S267/G270 and V170, respectively, although their relatively weak density indicates that these potential H-bonding interactions are not strong. The E171 is fully conserved in IDO1 family of enzymes (Supplementary Fig. 1). In addition, it is only one helical turn away from S167 in the B-Helix, which directly interacts with the Trp bound in the Sa site, suggesting that it is a structural element mediating the structural communication between the Si and Sa sites. Although both the IDE complex and the two Trp-bound complex of the F270G mutant exhibit impeded enzyme activity (Fig. 4a), their structures are surprisingly similar to that of the wild-type complex (Fig. 1), except that the heme is more planar and that the protein matrix is more flexible, in particular in the regions near the JK-Loop^C^, the K-Helix and D-Helix (Supplementary Fig. 6), as exemplified by the ability of the R231 sidechain to adopt two conformations in the IDE complex (Fig. 4b). The structural data suggest that the enzyme activity is controlled by protein dynamics, as well as heme distortion, which has been recognized as an important factor in tuning the functionalities of a variety of hemeproteins, including hIDO1^3,28–31^.

### Binding mode of mitomycin C

To determine if the Si site can be a target for allosteric inhibitors, we carried out molecular docking studies with an uncompetitive inhibitor, mitomycin C^25^. The data demonstrate that mitomycin C preferentially binds to the Si site (Fig. 4d), not the Sa site (see Methods section). In contrast to the much smaller IDE and Trp, it establishes extensive contacts with the protein matrix. Specifically, its quinone group interacts with the heme by *π*-stacking with it, while its polar groups are within H-bonding distances from V170, D274, G270, R343, and the 6-propionate group of the heme. The data support the view that identification of the Si site as a new inhibitor-binding site, as well as the disclosure of the unique binding modes of the substrate Trp and the inhibitor epacadostat in the Sa site, offer enticing prospects for structure-based designed of new generations of hIDO1 inhibitors. Furthermore, the fact that the Si site is only present in hIDO1, not in hTDO, (Supplementary Fig. 2) opens up new opportunities for rational design of hIDO1-selective inhibitors.

## Discussion

Dioxygenases belong to one of the three major classes of heme-based enzymes that utilize atmospheric O_2_ as a substrate^1^. Oxidases, such as cytochrome *c* oxidase, reduce O_2_ to two water molecules by consuming four electrons and four protons, and utilize the redox energy to pump four protons across the protein matrix. Monooxygenases, such as P450s, convert one atom of dioxygen to a water by using two electrons and two protons, and utilize the redox energy to insert the other oxygen atom into an organic substrate to generate an alcohol type of product. The dioxygenases, hIDO1, hIDO2, and hTDO, are unique as they insert both atoms of O_2_ into Trp without consuming any electrons or protons. The structural features of hIDO1 reported here offer an important blueprint for computational validation of the hypothesized radical addition mechanism (Supplementary Fig. 4) and provide new guidelines for structure-based design of hIDO1-selective inhibitors.

## Methods

### Protein preparation

The wild-type hIDO1 and F270G mutant proteins were expressed in *Escherichia coli* BL21 (DE3) cells^32^. The *E. coli* cells were grown in TB broth with 100 μg ml^−1^ ampicillin at 37 °C. When the O.D._600 nm_ of the culture reached ~0.8–1.0, the temperature was lowered to 25 °C; hemin chloride (8 μM), glucose (0.2%), and IPTG (1 mM) were then added to the culture to induce protein expression. The cells were grown for an additional 12–16 h, before they were harvested and lysed. The cell lysate was then loaded into a Ni-NTA column (Novagen). The target proteins were eluted with 150 mM imidazole in pH 7.4 Tris buffer (50 mM with 500 mM NaCl), and then oxidized by ferricyanide, to ensure a homogeneous population of the proteins in the ferric state. The mixture was loaded into a G25 column to eliminate NaCl, imidazole and ferricyanide, as well as its reduction product, and to exchange the buffer to pH 7.4 Tris buffer (50 mM). The purified proteins were stored at −80 °C until usage.

The F270G mutant was constructed by using the QuikChange II Site-Directed Mutagenesis Kit (Agilent, Santa Clara, CA) using the following primers (Sigma-Aldrich, St. Louis, MO):

5′-CAGGCCAAAGCAGCGTCGGTCAGTGCTTTGACGTCC-3′,

5′-GGACGTCAAAGCACTGACCGACGCTGCTTTGGCCTG-3′.

The plasmid DNA containing the wild-type *hIDO* gene in a pET100/D-TOPO vector (Invitrogen, Carlsbad, CA) was used as the template. The plasmid DNA was confirmed by DNA sequencing before it was transformed into *E. coli* BL21 (DE3) cells.

### Crystal preparation

The crystal growth was initiated by mixing protein solutions (40 mg ml^−1^) with precipitant solution (100 mM sodium thiosulfate in 100 mM CAPS buffer (pH 10.0) and 20% (w/v) PEG 8000). Crystals were grown at 4 °C using the microbatch method. The Trp complexes of the wild type and mutant hIDO1 were prepared with 10 mM and 40 mM Trp, respectively; while the IDE complexes were prepared with 2 mM and 5 mM Trp, respectively, in the presence of 10 mM IDE. The crystals of the hIDO1-epacadostat complex was prepared by soaking the hIDO1-CN-Trp crystals with 5 mM epacadostat dissolved in the crystallization solution. All crystals were cryoprotected by supplementing the mother solution with 20% (v/v) ethylene glycol before they were flash-frozen in liquid nitrogen for data collection.

### Crystallographic data collection and analysis

The data of all hIDO1 complexes, except the epacadostat complex, were collected remotely using the Stanford Synchrotron Radiation Lightsource (SSRL) beamline 9-2. The data of the epacadostat complex were collected by the Lilly Research Laboratories Collaborative Access Team (LRL-CAT) beamline staff at Sector 31 of the Advanced Photon Source. The diffraction images were indexed, integrated, and scaled with XDS^33^ and Aimless^34^. The Karplus–Diederichs method^35^ was used to find a proper resolution cutoff for each structure. Molecular replacement was conducted with Phaser^36^ through the CCP4i graphic interface^37^ using the 4-phenylimidazole complex structure (PDB code: 2D0T) as the search model. Further model building was performed using COOT^38^. Structure refinements were performed using Refmac5^37,39,40^. To confirm the pose of the bound epacadostat, single wavelength anomalous diffraction (SAD) data were collected at a wavelength near the bromine absorption edge (*λ* = 0.91988 Å). SAD data were processed using XDS and were scaled and merged using Aimless. Molecular replacement, model building, and refinement were carried out as described above. Omit phases from advanced stages of building and refinement (i.e., prior to inclusion of epacadostat) were used in a Fourier synthesis to generate an anomalous difference electron density map at 4.0 Å. The prominent peaks identified in this map were consistent with heme iron sites and bromine positions present in epacadostat allowing unambiguous positioning of the aryl moiety of the inhibitor. Data processing and refinement statistics are summarized in Table1. The structural models were displayed with PyMOL (http://www.pymol.org/), based on the subunit B, as its electron density is in general stronger than that of the subunit A.

**Table 1:** Crystallographic data collection and refinement statistics

Data set	hIDO1-CN-Trp	hIDO1-epacadostat	hIDO1-CN-Trp (IDE)^si^	F270G-CN-Trp (Trp)^si^	F270G-CN-Trp (IDE)^si^
**Data collection**					
PDBID	5WMU	5WN8	5WMV	5WMW	5WMX
X-ray source	SSRL 9-2	APS	SSRL 9-2	SSRL 9-2	SSRL 9-2
Wavelength (Å)	0.97945	0.91988	0.97945	0.97945	0.97945
Space group	P2_1_2_1_2_1_	P2_1_2_1_2_1_	P2_1_2_1_2_1_	P2_1_2_1_2_1_	P2_1_2_1_2_1_
Unit cell dimensions					
*a*, *b*, *c* (Å)	89.3, 97.8, 125.6	86.7, 97.8, 128.7	89.09, 97.7, 127.1	88.2, 97.9, 127.8	87.7, 97.6, 130.4
*α*, *β*, *γ* (°)	90, 90, 90	90, 90, 90	90, 90, 90	90, 90, 90	90, 90, 90
Resolution (Å)	38.70–2.40	29.69–2.50	38.80–2.60	39.08–3.03	39.06–2.69
No. of unique reflections	43,748 (4467)	38,582 (5556)	34,774 (4049)	21,897 (3840)	31,517 (4082)
*R*_merge_ (%)	4.6 (108)	8.7 (230)	6.7 (171)	8.7 (112)	6.8 (175)
*R*_pim_ (%)	2.2 (52)	2.3 (61)	3.0 (82)	4.2 (55)	3.3 (85)
I/*σ*(I)	17.4 (1.6)	22.5 (1.3)	15.5 (0.9)	14.1 (1.4)	12.8 (0.9)
CC_1/2_	1.00 (0.70)	1.00 (0.59)	1.00 (0.51)	1.00 (0.62)	1.00 (0.44)
CC_anomalous_	N/A	0.131	N/A	N/A	N/A
Completeness (%)	99.8 (98.3)	99.9 (100.0)	99.4 (95.9)	99.8 (98.5)	99.6 (98.3)
Redundancy	6.0 (6.0)	14.8 (14.9)	6.0 (6.1)	6.0 (5.9)	5.9 (6.0)
**Refinement**					
Resolution (Å)	38.65–2.40	29.69–2.50	38.88–2.60	39.08–3.03	39.06–2.69
No. of reflections	41,644 (2899)	36,596 (2640)	32,642 (2232)	19,850 (1380)	29,358 (2021)
*R*_work_/*R*_free_	0.2128/0.2560	0.2135/0.2518	0.2191/0.2591	0.2108/0.2587	0.2135/0.2526
No. of atoms					
Protein	6003	5880	6025	5998	6006
Ligand/ions	120	136	144	150	144
Water	224	148	158	104	131
B factor (mean) (Å^2^)	77.0	80.2	94.6	101.2	99.4
R.m.s. deviations					
Bond lengths (Å)	0.007	0.007	0.008	0.007	0.007
Bond angles (°)	1.08	1.20	1.19	1.02	1.05

Values in parenthesis are for highest resolution shell

### Activity measurements

The steady-state activities were measured in 50 mM Tris buffer (pH 7.4) at 25 °C with a standard protocol^25,41,42^. Briefly, the reactions were initiated by adding ascorbate into a solution mixture containing 0.1–0.25 μM protein, 12 μM methylene blue, 200 nM catalase, and a desired amount of L-Trp and/or IDE in 100 mM Tris (pH 7.4) in a quartz cuvette. The initial linear velocities of the reactions, V, were obtained by monitoring the formation of the product, NFK, at 321 nm (*ε* = 3750 M^−1^ cm^−1^) as a function of time with a UV2100 spectrophotometer (Shimadzu Scientific Instruments, Inc.) with a spectral slit width of 2 nm. V was calibrated with the concentration of the enzyme. IDE titration of the wild-type enzyme and the F270G mutant were carried out with 0.1 mM Trp. All the data were analyzed with Origin 6.1 software (Microcal Software, Inc., MA).

### Optical absorption spectroscopic measurements

The absorption spectra of hIDO1 in complex with various inhibitors were obtained with a UV2100 spectrophotometer (Shimadzu Scientific Instruments, Inc.) with a spectral slit width of 1 nm. The samples were prepared with 4 μM hIDO1 and 0.2–1.0 mM inhibitors in 50 mM Tris buffer (pH 7.4).

### Molecular docking studies

Molecular docking studies were conducted using the X-ray structure of the two Trp-bound complex of the F270G mutant, with the Trp removed from the Si site, as the receptor. The receptor was prepared for docking using the Protein Preparation Wizard suite implemented in Maestro version 11 (Schrödinger, LLC). Hydrogen atoms and bond orders were added to the initial coordinates, while water molecules were removed from the binding pocket. The structure was protonated based on pH = 7.0. The positions of the hydrogen atoms were optimized using the OPLS3 force field. The ligand, mitomycin C (MitoC), was built using 2D draw (Maestro, version 11; Schrödinger, LLC), and prepared as a single, low energy three-dimensional structure using LigPrep Module (LigPrep 2.6; Schrödinger, LLC), which checks for various ionization states, tautomers, stereochemistry, and ring conformation. The ligand structure was energy minimized using the MacroModel suite45 of Maestro (OPLS3 force field, default settings). Protonation states and possible tautomers were then generated at pH = 7.0 using the Epik program. Molecular docking was performed with Glide 7.4 (ligand–receptor docking software; Schrödinger, LLC). A grid box was set by covering the volume of the Si site region with the van der Waals radius scaling to 1.0 with a partial cutoff of 0.25 to soften the potential for nonpolar parts of receptor, where other atoms are free of scaling. Glide standard precision mode, where the ligand sampling was set flexible, was used for the docking. The best docked substrate conformation, representing 90% of the observed poses is shown in Fig. 4d. Comparable studies with a grid box set in the Sa site region show that MitoC does not bind to the Sa site.

### Data availability

Coordinates and structure factors for the reported crystal structures have been deposited in the Protein Data Bank under the accession codes 5WN8, 5WMU, 5WMV, 5WMW, and 5WMX. Other data that support the findings of this study are available from the corresponding author on reasonable request.

## Electronic supplementary material

Supplementary Information
