# Supplementary material for: Structural insights into substrate and inhibitor binding sites in human indoleamine 2,3-dioxygenase 1
Source: Nat Commun. 2017 Nov 22;8:1693. doi: 10.1038/s41467-017-01725-8 (PMC5700043; doi:10.1038/s41467-017-01725-8)
Supplement: Supplementary file 1 — Supplementary Information [file 41467_2017_1725_MOESM1_ESM.pdf]

```
                                          N-terminal Domain
hIDO1 NPQENLPDFY NDWMFIAKHL PDLIESGQLR ERVEKLNMLS IDHLTDHKSQ RLARLVLGCI TMAYVWGKGH
mIDO1 HPLVELPDAY SPWVLVARNL PVLIENGQLR EEVEKLPTLS TDGLRGHRLQ RLAHLALGYI TMAYVWNRGD
 hTDO ---------- ---------- ---------- ---------- ---------- ---------- ----------
 mTDO ---------- ---------- ---------- ---------- ---------- ---------- ----------
xcTDO ---------- ---------- ---------- ---------- ---------- ---------- ----------

                                 N-terminal Domain                           B
hIDO1 GDVRKVLPRN IAVPYCQLSK KLELPPILVY ADCVLANWKK KDPNKP-LT- YENMDVLFSF RDGDCS----
mIDO1 DDVRKVLPRN IAVPYCELSE KLGLPPILSY ADCVLANWKK KDPNGP-MT- YENMDILFSF PGGDCD----
 hTDO ---------- ---------- -----GGLIY GNYLH-LEKV LNA------- -----QELQS ETKGNKIHDE
 mTDO ---------- ---------- -----GGLIY GNYLQ-LEKI LNA------- -----QELQS EVKGNKIHDE
xcTDO ---------- ---------- ----EGRLTY GGYLR-LDQL LSA------- -----QQPLS ---EPAHHDE
                                   126    130

                   B                                   C                     D
hIDO1 KGFFLVSLLV EIAAASAIKV IPTVFKAMQM Q------ERD TLLKALLEIA SCLEKALQVF HQIHDHVNPK
mIDO1 KGFFLVSLLV EIAASPAIKA IPTVSSAVER Q------DLK ALEKALHDIA TSLEKAKEIF KRMRDFVDPD
 hTDO HLFIITHQAY ELWFKQILWE LDSVREIFQN GHVRDERNML KVVSRMHRVS VILKLLVQQF SILE-TMTAL
 mTDO HLFIITHQAY ELWFKQILWE LDSVREIFQN GHVRDERNML KVIARMHRVV VIFKLLVQQF SVLE-TMTAL
xcTDO MLFIIQHQTS ELWLKLLAHE LRAAIVHLQR D------EVW QCRKVLARSK QVLRQLTEQW SVLE-TLTPS
        163   167   171                                              214

     D                  DE-Hairpin               DE-Loop       E              F
hIDO1 AFFSVLRIYL SGWKGNPQLS DGLVYEGFWE DPKEFAGGSA GQSSVFQCFD VLLGIQQTAG GGHAAQFLQD
mIDO1 TFFHVLRIYL SGWKCSSKLP EGLLYEGVWD TPKMFSGGSA GQSSIFQSLD VLLGIKHEAG KESPAEFLQE
 hTDO DF-NDFREYL S--------- ---------- ------PASG FQSLQFRLLE NKIGVLQNMR VPYNRKHYRD
 mTDO DF-NDFREYL S--------- ---------- ------PASG FQSLQFRLLE NKIGVLQSLR VPYNRKHYRD
xcTDO EY-MGFRDVL G--------- ---------- ------PSSG FQSLQYRYIE FLLGNKNP-- -----Q-MLQ
        231                                 264      270   274

    F          G            H
hIDO1 MRRYMPPAHR NFLCSLESNP SVREFVLS-- ---------- ---------- ---------- ----------
mIDO1 MREYMPPAHR NFLFFLESAP PVREFVIS-- ---------- ---------- ---------- ----------
 hTDO NF---KGEEN ELLLKSEQEK TLLELVEAWL ERTPGLEPHG FNFWGKLEKN ITRGLEEEFI RIQAKEESEE
 mTDO NF---GGDYN ELLLKSEQEQ TLLQLVEAWL ERTPGLEPNG FNFWGKFEKN ILKGLEEEFL RIQAKTDSEE
xcTDO VFAY-DPAGQ ARLREVLEAP SLYEEFLRYL ARFGHAIPQQ Y--------- ---------- ----------

                                                     I                   J
hIDO1 ---------- ---------- ---------- ---------- ---------- ------KGDA GLREAYDACV
mIDO1 ---------- ---------- ---------- ---------- ---------- ------RHNE DLTKAYNECV
 hTDO KQEQVAEFQK QKEVLLSLFD EKRHEHLL-S KGERRLSYRA LQGALMIYFY REEPRFQVPF QLLTSLMDID
 mTDO KEEQMAEFRK QKEVLLCLFD EKRHDYLL-S KGERRLSYRA LQGALMIYFY REEPRFQVPF QLLTSLMDID
xcTDO ---------- ---------- ---QARDWTA AHV--ADDTL RPVFERIYEN T--DRYWREY SLCEDLVDVE

               J                 JK-Loop^N          JK-Loop^C         K
hIDO1 KALVSLRSYH LQIVTKYILI PASQQPKENK TSEDPSKLEA KGTGGTDLMN FLKTVRS--T TEKSLLKEG-
mIDO1 NGLVSVRKFH LAIVDTYIMK PSKKKPTDGD KSEEPSNVES RGTGGTNPMT FLRSVKD--T TEKALLSWP-
 hTDO SLMTKWRYNH VCMVHRML-- ---------- -------GSK AGTGGSSGYH YLRSTVSDRY KVFVDLFNLS
 mTDO TLMTKWRYNH VCMVHRML-- ---------- -------GTK AGTGGSSGYH YLRSTVSDRY KVFVDLFNLS
xcTDO TQFQLWRFRH MRTVMRVI-- ---------- -------GFK RGTGGSSGVG FLQQALA--L TFFPELFDVR
            343  346                             378     379     387

hIDO1 --------
mIDO1 --------
 hTDO TYLIPRHWIP KMNPWW
 mTDO TYLVPRHWVP KMNPIIHKFL YTAEYSDSSY FSSDESD
xcTDO TSVGVWW
```

Supplementary Figure 1. Structure-based sequence alignment of the IDO1 and TDO families of enzymes. The nomenclature of the helices is indicated on the top. The sequences of mIDO and mTDO are taken from the mouse homologs, while that of xcTDO is taken from the *Xanthomonas campestris* homolog. The fully and partially conserved residues are labeled in red and blue, respectively. The blue double arrow indicates the conserved N-terminal fragment. The residue numbers shown at the bottom indicate critical residues of hIDO1 mentioned in the text. The residues subjected to posttranslational modifications are labeled in magenta. The alignment was obtained using Promals3D (http://prodata.swmed.edu/promals3d/).

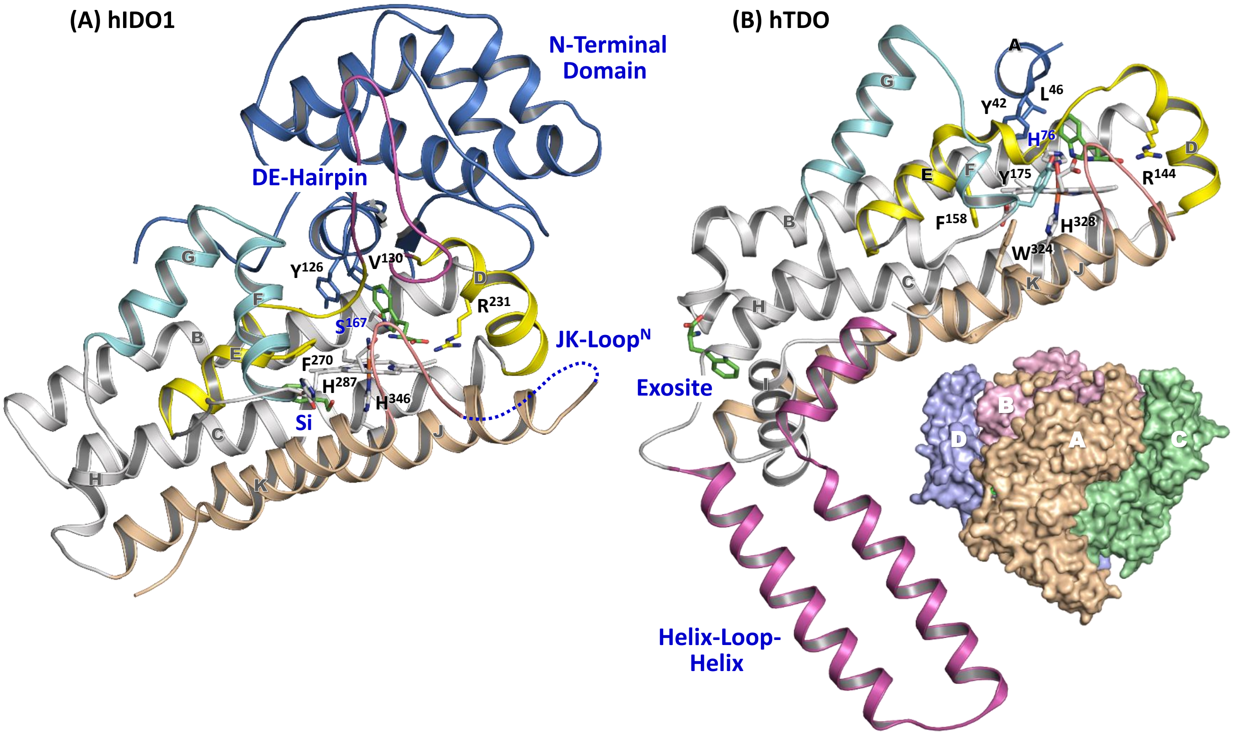

Supplementary Figure 2. Structural comparison of hIDO1 with hTDO. The structures of hIDO1-CN-Trp complex (A) and hTDO-O_2-Trp complex (B) are taken from this work and literature[1] (PDB code: 5TI9), respectively. hIDO1 shares high structural similarity with hTDO, but with several prominent exceptions. (1) hIDO1 is a monomer, while hTDO is a tetramer made by a dimer of dimers, see the inset in (B). (2) In hIDO1, the indoleamine of the substrate Trp forms water-mediated H-bonds with S167; while that in hTDO forms a direct H-bond with H76. (3) hIDO1 contains a small N-terminal domain (colored in blue), a long DE-hairpin insertion in the DE-Loop (colored in magenta), and a highly flexible JK-Loop^N (shown as the blue dotted line), which are only present in hIDO1, not in hTDO; conversely, hTDO contains an additional I-Helix, and a long helix-loop-helix domain insertion between the H-Helix and I-Helix (colored in magenta), which is only present in hTDO, not in hIDO1. (4) The top of the Trp binding pocket in hIDO1 is sealed by a small helix from the N-terminal domain; while that in hTDO is covered by a comparable A-Helix from the neighboring subunit (colored in blue), *via* domain swapping. (5) The second Trp binding site, Si, found in hIDO1 is not functional in hTDO, as it is partially occupied by the sidechains of F158 (equivalent to F270 in hIDO1) and W324; in contrast, hTDO also binds a second Trp, but it is located in a distinct exosite, ~42 Å away from the Sa site, formed by residues fully conserved in TDOs from Trp auxotrophic species.[1] The distinct second Trp binding sites in the two enzymes are plausibly important for the regulation of their *in vivo* activities. hTDO is a constitutive enzyme in the liver, which plays a critical role in regulating systemic Trp levels. Trp binding to the exosite in hTDO does not directly affect the enzyme activity; instead it regulates the cellular lifetime of the enzyme through the ubiquitin-dependent proteasomal pathway,[1] which is conceivably pivotal for maintaining normal Trp homeostasis. In contrast, hIDO1 is an inducible enzyme, whose concentration can be raised very high upon stimulation. Trp binding to the Si site in hIDO1 negatively regulates the enzyme activity, which is plausibly critical for cell survival by limiting the synthesis of the kynurenine pathway metabolites (which can be hazardous to cells), in particular when large [Trp] fluctuations arise during meals. A group of ubiquitination sites are identified in the helix-loop-helix domain in hTDO,[1] while posttranslational phosphorylation sites are found in the N-terminal domain,[2-4] the DE-hairpin motif,[2-4] and the JK-Loop^N region[5] in hIDO1, indicating that the characteristic structural features of hIDO1 and hTDO are important for their specific *in vivo* functions.

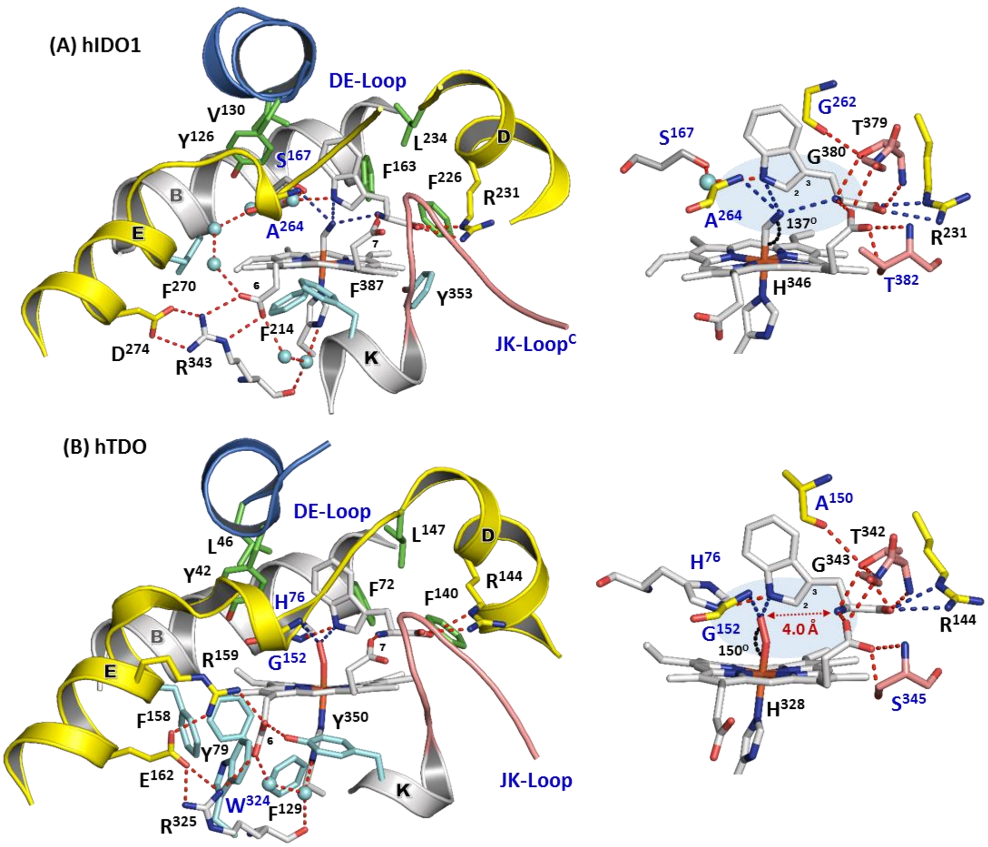

Supplementary Figure 3. Comparison of the active site structure of hIDO1 with that of hTDO. The structure of hIDO1 (A) is duplicated from Figure 1B-C. The structure of hTDO (B) is based on the O_2 and Trp bound ferrous complex taken from literature[1] (PDB code: 5TI9). In hIDO1, the heme is stabilized by hydrophobic interactions with F270, F214, F387 and Y353 (colored in cyan). Its 7-propionate group establishes an extended H-bonding network with the ammonium group of the Trp and several residues in the JK-Loop^{C} and DE-Loop, while the 6-propionate group forms an extended H-bonding network with the D274/ R343 ion pair and H346 (*via* two intervening water molecules). In hTDO, more extensive hydrophobic interactions between the heme and the protein matrix are evident; the imidazole ring of the proximal histidine rotates by $\sim 30^{o}$; in addition, the proximal H-bonding network involving the 6-propionate group of the heme is more widespread, due to the participation of R159 and Y350, in addition to the E162/R325 ion pair that is equivalent to the R343/D274 ion pair in hIDO1. In hIDO1, the bound Trp is stabilized by hydrophilic and hydrophobic interactions with the heme and the protein matrix as discussed in the main text. In hTDO, similar interactions are observed, but with several noticeable exceptions. In particular, (1) H76 replaces S167 to form a direct H-bond with the indoleamine group of the Trp, and (2) the Fe-O-O moiety bends away from the ammonium group of the Trp, preventing direct H-bonding interaction between them. Previous QM/MM studies[6] suggest that the ammonium group of the Trp is important in positioning heme iron-bound dioxygen for its insertion into the C_2 of Trp (see Supplementary Figure 4). This hypothesis is supported by the current hIDO1-CN-Trp structure, but not the reported hTDO-O_2-Trp structure,[1] hinting that variations may exist in the substrate activation mechanisms of the two human heme-based dioxygenases (although in both cases the iron-bound ligand is positioned closer to C_2, rather than C_3, consistent with the radical addition mechanism described in Supplementary Figure 4).

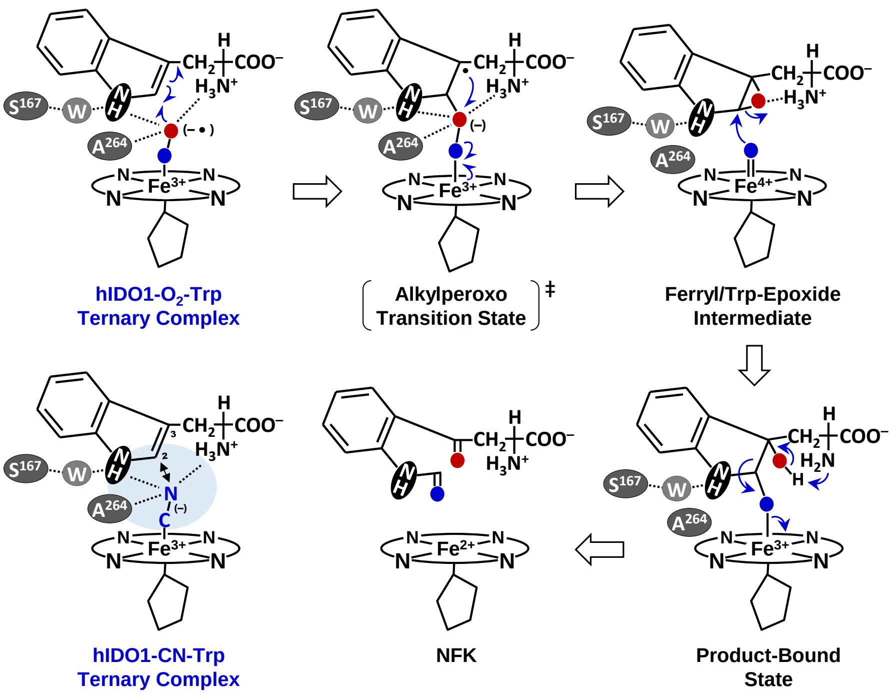

Supplementary Figure 4. Comparison of the hIDO1-CN-Trp complex structure with the hypothesized hIDO1-O_2-Trp complex structure derived from QM/MM simulations. The hIDO1-CN-Trp structure was taken from Figure 1. The hIDO1-O_2-Trp structure was taken from the previously proposed two-step ferryl-based dioxygenase mechanism (also known as "radical addition mechanism") derived from QM/MM simulations.[6,7,8] Based on that mechanism, the dioxygenase reaction is initiated by radical addition of the iron-bound dioxygen to the C_2 of the Trp to generate the ferryl and Trp-epoxide intermediate via the alkylperoxo transition state. The Trp-NH_3^+ then protonates the epoxide ring, thereby triggering the addition of the ferryl oxygen to the C_2 to generate NFK. Resonance Raman studies indicate that the iron-bound dioxygen has ferric superoxide character,[6] supporting the view that the CN is a good surrogate for the heme iron-bound dioxygen. It is important to note that, in addition to the aforementioned radical addition mechanism, an alternative electrophilic addition mechanism has been proposed.[9,10] Based on that mechanism, the reaction is initiated by dioxygen addition to the C_3, the most electron rich position in the indole ring, in contrast to the electrophilic addition mechanism, which prefers O_2 addition to the C_2.[6-8,11,12] The current hIDO1-CN-Trp structure, as well as the previously reported hTDO-O_2-Trp structure,[1] is hence in better agreement with the radical addition mechanism.

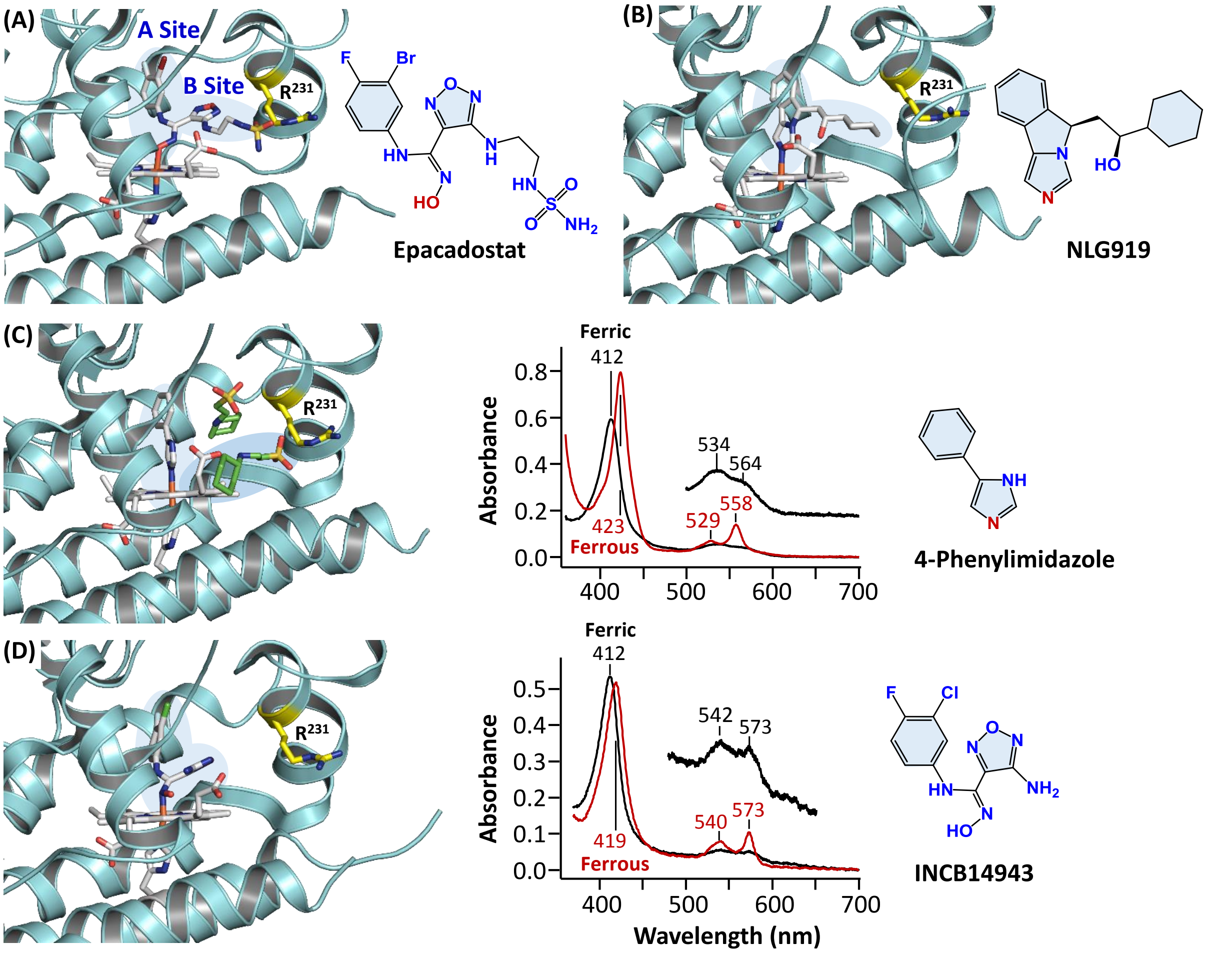

Supplementary Figure 5. Comparison of the binding modes of hIDO1 inhibitors. The structure of hIDO1-epacadostat complex (A) is obtained from this work, while those of hIDO1 in complex with NLG919 (B), 4-phenylimidazole (C) and INCB149439 (an analog of epacadostat) (D) are taken from literature with PDB codes of 5EK3[13], 2D0T[14] and 5XE1,[15] respectively. The areas highlighted in blue backgrounds are the so-called "A site" and "B site" respectively.[16] In all the inhibitor-bound states, the JK-Loop is entirely disordered; in addition, R231 swings out towards the bulk solvent. In the 4-phenylimidazole complex (C), the inhibitor occupies the A site, while one of the two CHES buffer molecules (colored in green) co-crystalized with the complex occupies the B site. NLG919 and 4-phenylimidazole coordinate to the heme iron *via* the nitrogen atom of their imidazole groups; in contrast, epacadostat coordinates to the heme iron *via* the oxygen atom of its hydroxyamidine group. The binding mode of INCB149439 (D) remains to be further investigated. The similarity of its optical absorption spectra (shown in the right panel) to those of the epacadostat complex (Figure 2B) suggests that INCB149439 coordinates to the heme iron via the oxygen atom of the hydroxyamidine group; however in the reported crystal structure it was modeled with the nitrogen atom of the hydroxyamidine group coordinating to the heme iron.[15] The low resolution of the complex structure (3.2 Å),[15] as well as the weak electron density of the bound inhibitor, plausibly hindered precise determination of the binding mode, calling for reexamination of the structure.

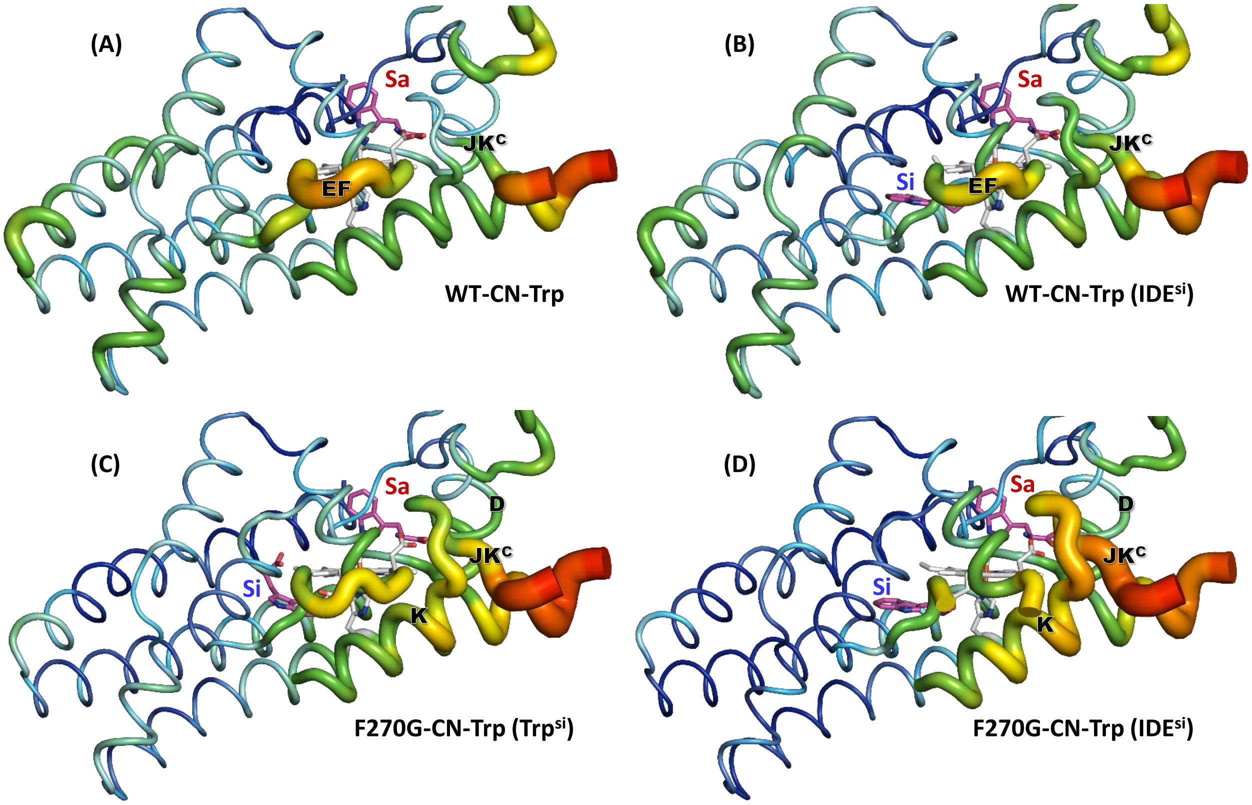

Supplementary Figure 6. Perturbation in the protein flexibility of hIDO1 by the F270G mutation and/or the occupation of the Si site by Trp or IDE. (A) and (B) show the structures of the wild type hIDO1 in the absence or presence IDE in the Si site, respectively. (C) and (D) show the structures of the F270G mutant with the Si site occupied by Trp and IDE, respectively. The structures are presented in B-factor putty mode, in which the more flexible regions are displayed with larger thickness and colored red. For clarity only the residues [161-395] are shown. The bound Trp or IDE molecules are shown as magenta sticks. The structures of the JK-Loop^{N} in all structures, as well as a part of the EF-Loop in (D), are too flexible to be determined.

Supplementary References

1 Lewis-Ballester, A. *et al.* Molecular basis for catalysis and substrate-mediated cellular stabilization of human tryptophan 2,3-dioxygenase. *Sci Rep* **6**, 35169, doi:10.1038/srep35169 (2016).

2 Orabona, C., Pallotta, M. T. & Grohmann, U. Different partners, opposite outcomes: a new perspective of the immunobiology of indoleamine 2,3-dioxygenase. *Mol Med* **18**, 834-842, doi:10.2119/molmed.2012.00029 (2012).

3 Orabona, C. *et al.* SOCS3 drives proteasomal degradation of indoleamine 2,3-dioxygenase (IDO) and antagonizes IDO-dependent tolerogenesis. *Proc Natl Acad Sci U S A* **105**, 20828-20833, doi:10.1073/pnas.0810278105 (2008).

4 Pallotta, M. T. *et al.* Indoleamine 2,3-dioxygenase is a signaling protein in long-term tolerance by dendritic cells. *Nat Immunol* **12**, 870-878, doi:10.1038/ni.2077 (2011).

5 Alvarez, L. *et al.* Structural Study of a Flexible Active Site Loop in Human Indoleamine 2,3-Dioxygenase and Its Functional Implications. *Biochemistry* **55**, 2785-2793, doi:10.1021/acs.biochem.6b00077 (2016).

6 Lewis-Ballester, A. *et al.* Evidence for a ferryl intermediate in a heme-based dioxygenase. *Proc Natl Acad Sci U S A* **106**, 17371-17376, doi:10.1073/pnas.0906655106 (2009).

7 Capece, L. *et al.* The first step of the dioxygenation reaction carried out by tryptophan dioxygenase and indoleamine 2,3-dioxygenase as revealed by quantum mechanical/molecular mechanical studies. *J Biol Inorg Chem* **15**, 811-823, doi:10.1007/s00775-010-0646-x (2010).

8 Capece, L., Lewis-Ballester, A., Yeh, S. R., Estrin, D. A. & Marti, M. A. Complete reaction mechanism of indoleamine 2,3-dioxygenase as revealed by QM/MM simulations. *J Phys Chem B* **116**, 1401-1413, doi:10.1021/jp2082825 (2012).

9 Raven, E. L. A short history of heme dioxygenases: rise, fall and rise again. *J Biol Inorg Chem* **22**, 175-183, doi:10.1007/s00775-016-1412-5 (2017).

10 Makino, R. *et al.* Initial O(2) Insertion Step of the Tryptophan Dioxygenase Reaction Proposed by a Heme-Modification Study. *Biochemistry* **54**, 3604-3616, doi:10.1021/acs.biochem.5b00048 (2015).

11 Chung, L. W., Li, X., Sugimoto, H., Shiro, Y. & Morokuma, K. ONIOM study on a missing piece in our understanding of heme chemistry: bacterial tryptophan 2,3-dioxygenase with dual oxidants. *Journal of the American Chemical Society* **132**, 11993-12005, doi:10.1021/ja103530v (2010).

12 Chung, L. W., Li, X., Sugimoto, H., Shiro, Y. & Morokuma, K. Density functional theory study on a missing piece in understanding of heme chemistry: the reaction mechanism for indoleamine 2,3-dioxygenase and tryptophan 2,3-dioxygenase. *J Am Chem Soc* **130**, 12299-12309, doi:10.1021/ja803107w (2008).

13 Peng, Y. H. *et al.* Important Hydrogen Bond Networks in Indoleamine 2,3-Dioxygenase 1 (IDO1) Inhibitor Design Revealed by Crystal Structures of Imidazoleisoindole Derivatives with IDO1. *J Med Chem* **59**, 282-293, doi:10.1021/acs.jmedchem.5b01390 (2016).

14 Sugimoto, H. *et al.* Crystal structure of human indoleamine 2,3-dioxygenase: catalytic mechanism of O2 incorporation by a heme-containing dioxygenase. *Proc Natl Acad Sci U S A* **103**, 2611-2616 (2006).

15 Wu, Y., Xu, T., Liu, J., Ding, K. & Xu, J. Structural insights into the binding mechanism of IDO1 with hydroxylamidine based inhibitor INCB14943. *Biochem Biophys Res Commun* **487**, 339-343, doi:10.1016/j.bbrc.2017.04.061 (2017).

16 Rohrig, U. F., Majjigapu, S. R., Vogel, P., Zoete, V. & Michielin, O. Challenges in the Discovery of Indoleamine 2,3-Dioxygenase 1 (IDO1) Inhibitors. *J Med Chem* **58**, 9421-9437, doi:10.1021/acs.jmedchem.5b00326 (2015).
